# Selling World Health Organization's Alcohol “Best Buys” and Other Recommended Interventions in an Urban Chinese Population: Public Acceptability of Alcohol Harms Reduction Strategies in Hong Kong

**DOI:** 10.3389/fpubh.2022.855416

**Published:** 2022-04-21

**Authors:** Jiazhou Yu, Dong Dong, Timothy S. Sumerlin, William B. Goggins, Qi Feng, Jean H. Kim

**Affiliations:** ^1^Jockey Club School of Public Health and Primary Care, The Chinese University of Hong Kong, Shatin, Hong Kong SAR, China; ^2^Centre for Health Systems and Policy Research, The Chinese University of Hong Kong, Shatin, Hong Kong SAR, China; ^3^Nuffield Department of Population Health, University of Oxford, Oxford, United Kingdom

**Keywords:** public opinion, acceptability, perception, alcohol, policy, Chinese, survey

## Abstract

**Background:**

To counter the harms caused by alcohol use, the World Health Organization (WHO) outlined a series of evidence-based recommendations, including the highly cost-effective “Best Buys” recommendations. While many Western countries have been actively introducing alcohol harms reduction strategies, it is unclear whether these cost-effective policies would be publicly acceptable in Asian regions with traditionally low alcohol consumption. This study examines the public acceptability of WHO-recommended alcohol harms reduction strategies in an Asian city with few extant alcohol regulations.

**Methods:**

A cross-sectional telephone survey of Hong Kong Chinese residents aged 18–74 (*n* = 4,000) was conducted from January to August 2018. Respondents were asked about their perceptions of various WHO-recommended strategies and consequences of their implementation. After reducing the strategies into several policy categories by principal component analysis, multivariable linear regression was performed to identify factors associated with endorsement of the various policies.

**Results:**

Among the “Best Buys”, introduction of moderate beer/wine taxes (68.7%) and shortened alcohol retail hours (51.9%) were the most supported while bans on event sponsorships (19.5%) and public drinking events (17.7%) were the least popular. Strategies targeting young drinkers were particularly highly supported. Males, younger adults, Non-abstainers, and those who believed in drinking's social benefits were less likely to endorse stringent control measures (*p* < 0.05). Adults with higher household income were less supportive, partially due to concerns about infringements on local economy, lifestyles, and economic freedom. Women and older people were generally more supportive, partially because they perceived these policies would lower alcohol-related harms.

**Conclusion:**

In order to reduce barriers to implementing WHO-recommended strategies in the region, it is imperative to increase awareness of alcohol-related harms and to strengthen beliefs in the effectiveness of these countermeasures, especially among men, young adults, and drinkers.

## Introduction

Excessive alcohol use ranks among the top risk factors for disease, disability, and death throughout the world, and is correlated with a wide range of social harms such as interpersonal violence, impaired work performance, and domestic problems (1). In response to the well-noted harms of alcohol use, the World Health Organization (WHO) outlined a set of comprehensive, evidence-based recommendations, including: 1) the most cost-effective “Best Buys” policy actions which call for increasing alcoholic beverage excise taxes, restricting access to retailed alcoholic beverages, and comprehensive advertising, promotion, and sponsorship bans; 2) other interventions related to drink-driving, minimum pricing, minimum purchase age, sponsorship restriction, information provision, and interventions targeting harmful drinking (2). Although policy decisions are largely based upon evidence of cost-effectiveness, public acceptability is also an important but comparatively under examined consideration. Greater public acceptability can reduce enforcement costs, increase policy sustainability as well as improve community involvement and compliance.

Previous studies, predominantly conducted in Western countries, have noted that public acceptability of alcohol policies varies by the policy nature and by respondents' characteristics. Although proven to be effective in evaluations, restrictive policies designed to affect behaviors (e.g., limiting access to alcohol) are, in general, less popular than policies that are less intrusive (e.g., educational measures) (3–5). Policies that focus on young people or target certain groups of drinkers are consistently more supported than population-wide measures that potentially affect all drinkers (6–8). Studies across different settings have reported that women, older adults, and abstainer or light drinkers are significantly more supportive of a range of alcohol policies than men, younger people, and frequent or heavy drinkers (7, 9–15). Fewer studies have investigated the effect of socio-economic status, with generally inconsistent results reported (13, 16–19). Moreover, perception of alcohol use in general and of specific regulations could potentially mediate the individual's attitudes toward alcohol policy. For example, awareness or experience of harms from alcohol (18, 20, 21), beliefs about the negative consequences of drinking behaviors (22), and greater perceived strategy effectiveness (7, 23) have been found to be associated with greater support for alcohol control measures.

The preponderance of the existing evidence comes from high-income, Western countries with notably high alcohol use levels, high prevalence of alcohol-related harms (24–26), and stringent alcohol policies already in place. Acceptability of policies designed to reduce alcohol-related harms such as random breath testing and restricted retail sale hours, have often been debated in terms of infringements on citizen's civil liberties (27, 28). In contrast, public acceptability toward alcohol policies remains largely unclear in Asian regions, including Hong Kong which is characterized as a region with much lower levels of civil liberties than Western European countries, the US, and Australia where alcohol policies are often publicly debated (29). Hong Kong is currently an autonomous, special administrative region of China with a westernized legal system, liberal capitalist economy, and free trade policies distinct from those of Mainland China, allowing for rapid expansion of alcohol markets. Although the alcohol consumption level in Hong Kong is historically lower than that of Western countries and some Asian countries such as Japan and South Korea (1, 30–32), alcohol is commonly consumed in festive occasions and for social purposes (33). A population-based survey has revealed that alcohol-related harms were commonplace in public settings, and one-fifth of the Hong Kong population had been adversely affected by others' drinking on various occasions, involving harms to their health, work, and interpersonal relationship. Young people and heavy drinkers are noted to be at high risk of different types of alcohol-related harms (34). While many national governments have been increasing alcohol taxes to reduce alcohol-related harms, Hong Kong eliminated the 20% tax on beer and 40% tax on wine in 2008 as part of a strategic plan to make the city a regional alcohol trading center (35). This move has made Hong Kong one of the very few places in the world where beer and wine are completely untaxed. Subsequent increases were observed in the importation and consumption of alcohol, (30, 36) with concomitant upsurge in the number of liquor licenses (37–39). Despite this, Hong Kong currently possesses no restriction on time and place of alcohol sales or the density of alcohol outlets; alcohol promotion/advertising through social media and sponsorship are completely unregulated; there is currently no minimum pricing regulation or mandatory warning labels on alcohol containers. Although minimum alcohol purchasing age of 18 was enacted in 2018, the regulation has been noted to be under-enforced (40). Apart from stringent drink-driving laws which significantly reduced drinking-related traffic crashes (41) and brief intervention services provided in selected hospitals (42), the scope of Hong Kong's alcohol control policies falls short of the WHO recommended strategies, especially the “Best Buys” on alcohol taxation, availability, and advertising. Although implementation of WHO recommendations would likely curb the increasing trend of alcohol use and subsequent harms, it is unclear whether public acceptability for adopting various alcohol harms reduction policies would be comparable to Western countries such as the US in a region with low alcohol consumption culture and few regulatory measures.

To provide an evidence base for devising publicly-supported regulatory actions, this population-based survey examined the public acceptability and perceived consequences of various alcohol harms reduction strategies in Hong Kong. These policies include strategies that are based on WHO's “Best Buys” as well as other commonly promoted recommendations. This study also attempts to identify the socio-demographic, attitudinal, and drinking-related factors associated with endorsement of various strategies, as well as the mediating effect of perception of policy consequences. The study results can help identify knowledge gaps in public understanding of alcohol-related issues and inform alcohol policy-making in Hong Kong as well as other economically-developed Asian regions.

## Methods

Chinese Hong Kong permanent residents were the target population of this study. An anonymous cross-sectional telephone survey using a structured questionnaire was conducted from January to August, 2018 in Cantonese. Random telephone numbers were selected from up-to-date telephone directories, which is a comprehensive list of landline numbers. For unanswered calls, four other independent calls were made at different times before considering the number to be invalid. The eligible individual whose birthday was the closest to the date of interview was invited to join the study. Verbal informed consent was obtained from respondents after assurances of anonymity. The response rate (completed interviews divided by the number of households contacted) was 62.5%. A final sample of 4,000 respondents was recruited and included in data analysis. Ethical approval was obtained from Ethics Committee of The Chinese University of Hong Kong.

### Measurements

The study instrument asked the respondents for their socio-demographic information (Table 1). Respondents who reported ever drinking a full alcohol serving (a can of beer/a glass of wine/a shot of spirits) were classified as ever drinkers. Those who did so in the past 12 months were classified as past-year drinkers and those who had consumed at least five alcohol servings on one occasion in the preceding 30 days were classified as past-month binge drinkers. Respondents who reported drinking at least once a week were classified as weekly drinkers. General attitudes toward alcohol use were assessed by asking respondents whether they think (yes/no): “alcohol is a public health issue in Hong Kong”, “habitual drinking is bad for health”, “occasional drinking is good for health”, “not knowing how to drink is bad for work”, and “drinking has noticeable social benefits”.

**Table 1:** Background characteristics of the study sample (*n* = 4,000).

	**Males (*n* = 1,737)**	**Females (*n* = 2,263)**		**Total (*n* = 4,000)**	**Hong Kong population^**a**^**
Age (years)	**% (** ***n*** **)**	**% (** ***n*** **)**	***P*** **(** **χ^2^)**	**% (** ***n*** **)**	**%**
18–24	11.8 (205)	10.8 (245)	0.96	11.3 (450)	10.9
25–34	15.3 (265)	15.9 (359)		15.6 (624)	17.6
35–44	17.1 (297)	16.5 (374)		16.8 (671)	18.5
45–54	19.9 (345)	20.5 (463)		20.2 (808)	21.4
55–64	22.2 (386)	23.1 (522)		22.7 (908)	20.4
65–74	13.8 (239)	13.3 (300)		13.5 (539)	11.3
Marital status					
Currently married	64.9 (1,127)	69.9 (1,582)	0.002	67.7 (2,709)	61.1
Single, never married	33.7 (586)	28.6 (648)		30.9 (1,234)	30.4
Divorced/Widowed/Separated	1.0 (18)	1.2 (26)		1.1 (44)	8.5
Education					
Secondary or less	54.4 (937)	57.2 (1,288)	<0.001	56.0 (2,225)	65.1
Upper secondary Non-degree	7.1 (123)	7.0 (159)		7.1 (282)	11.4
University or above	38.1 (662)	35.6 (805)		36.7 (1,467)	23.5
Dependent children <18 years old					
No	81.0 (1,407)	79.1 (1,789)	0.18	79.9 (3,196)	NA
Yes	18.3 (318)	19.9 (451)		19.2 (769)	NA
Employment					
Employed at least part-time	67.7 (1,173)	43.9 (990)	<0.001	54.2 (2,163)	59.0
Homemaker	0.4 (6)	37.4 (846)		21.3 (852)	10.1
Unemployed	1.7 (29)	1.1 (25)		1.4 (54)	2.1
Full-time student	8.1 (114)	7.0 (158)		7.5 (299)	7.1
Retired	22.1 (383)	10.3 (234)		15.4 (617)	17.8
District of residence					
Hong Kong Island	19.1 (332)	18.2 (412)	0.03	18.6 (744)	16.4
Kowloon	32.1 (558)	28.9 (655)		30.2 (1,213)	30.7
New Territories	47.2 (820)	49.8 (1,127)		48.7 (1,947)	52.9
Monthly household income					
<25,000 HKD	34.5 (599)	38.8 (877)	<0.001	36.9 (1,476)	47.2
25,000–49,999 HKD	36.4 (633)	29.0 (657)		32.2 (1,290)	29.1
≥50,000 HKD	11.1 (193)	12.2 (275)		11.7 (468)	23.7
Drinking patterns					
Ever drinking	57.5 (999)	36.7 (830)	<0.001	45.7 (1,829)	NA
Past-year drinking	44.3 (769)	28.7 (649)	<0.001	35.5 (1,418)	NA
Binge drinking^b^	10.0 (174)	5.0 (112)	0.01	7.2 (286)	NA
Weekly drinking^c^	10.2 (177)	3.6 (82)	<0.001	6.5 (259)	NA

*Totals for all categories may not sum to 4,000 due to missing data; NA, data not available.*

*^a^Based on calculations from 2016 Population By-census.*

*^b^Having consumed five or more drinks on one occasion in the past 30 days.*

*^c^Having consumed alcohol at least once a week in the past year*.

Based on the WHO's “Best Buys” and other recommendations for reducing alcohol-related harms, the authors first compiled a list of measures that are commonly implemented in other regions. Then, 17 policies that are most relevant to the current policy gap of Hong Kong were included in the instrument under four main categories to assess the public acceptability (Table 2). Respondents were asked whether they support (support/no opinion/against): 1) tax increases: introducing a 30% beer/wine tax; implementing a moderate (5–10%) beer/wine tax; 2) restriction of retailed alcohol availability: introducing “last orders” in bars; reducing retailed alcohol sale time; limiting location of alcohol establishments; 3) advertisement restrictions: banning alcohol advertising on TV, radio, and magazines; regulating social media advertisement; banning large alcohol advertisement on public billboards/public transport; and 4) other regulations: ID request for alcohol purchases in stores/restaurants and bars; minimum alcohol pricing; restricting number of alcohol serving establishments outside of tourist areas; banning alcohol event sponsorship; restricting high publicity drinking events; promoting alcohol-related education; mandating health warning labels; enforcing random breath test; promoting services for problem drinkers.

**Table 2:** Endorsement of various alcohol harms reduction strategies, by past-year drinking status (*n* = 4,000).

**Strategies**	**Supporting respondents %**			
	**Past-year drinkers**,	**Past-year abstainers**,		**Overall**,
	***n* = 1418**	***n* = 2582**		***n* = 4000**
***“Best Buys”***	***% (95% CI)***	***% (95% CI)***	***P(** **χ^2^)***	***% (95% CI)***
**Increasing taxes**				
Implementing a moderate beer and wine tax (e.g., 5–10%)	57.6% (55.0–60.2)	74.6% (72.9–76.3)	<0.001	68.7% (67.2–70.1)
Re-introducing a heavy 30% beer and wine tax	24.8% (22.6–27.1)	50.4% (48.5–52.4)	<0.001	41.4% (39.9–43.0)
**Restricting physical availability of retailed alcohol**				
Convenience stores not being permitted sell alcohol after a certain time	35.5% (33.0–38.0)	60.7% (58.8–62.6)	<0.001	51.9% (50.3–53.4)
Introducing “Last Order Times” in bars	30.0% (27.7–32.5)	52.2% (50.3–54.2)	<0.001	44.5% (42.9–46.0)
**Restricting exposure to alcohol advertising**				
Banning large alcohol advertisements on public billboards and public transport	23.7% (21.5–25.9)	40.9% (39.0–42.8)	<0.001	34.9% (33.4–36.4)
Banning all alcohol advertising on TV, radio, and magazines	21.5% (19.5–23.8)	41.1% (39.2–43.0)	<0.001	34.2% (32.8–35.7)
Greater social media regulation of alcohol advertisements	26.2% (24.0–28.6)	36.5% (34.7–38.4)	<0.001	32.9% (31.4–34.4)
***Other WHO-recommended strategies***				
More alcohol-related education especially for young people	93.5% (92.0–94.6)	97.6% (96.9–98.1)	<0.001	96.1% (95.5–96.7)
Enforcement of current random breath testing of drivers	88.8% (87.1–90.4)	94.4% (93.4–95.2)	<0.001	92.4% (91.6–93.2)
Drinking age verification at stores	79.1% (76.9–81.2)	86.0% (84.6–87.3)	<0.001	83.6% (82.4–84.7)
Increasing awareness of programmes like AA for problem drinkers	75.3% (73.0–77.5)	87.6% (86.2–88.8)	<0.001	83.3% (82.1–84.4)
Drinking age verification at bars and restaurants	76.3% (74.1–78.5)	84.1% (82.6–85.4)	<0.001	81.4% (80.1–82.6)
Mandatory health warning labels on alcoholic beverages and advertisements	59.2% (56.6–61.7)	76.3% (74.6–77.9)	<0.001	70.3% (68.8–71.7)
Limiting the number of alcohol serving establishments outside of the tourist areas	25.2% (23.0–27.5)	49.1% (47.1–51.0)	<0.001	40.7% (39.2–42.3)
Setting a minimum alcohol price	27.3% (25.1–29.7)	37.3% (35.4–39.2)	<0.001	33.7% (32.3–35.2)
Banning alcohol event sponsorship	14.4% (12.7–16.4)	22.3% (20.7–23.9)	<0.001	19.5% (18.3–20.8)
Restricting high publicity drinking events	9.7% (8.3–11.4)	22.1% (20.5–23.7)	<0.001	17.7% (16.5–18.9)

Prior to the commencement of the study, the authors conducted a focus group where drinking (*n* = 5) and Non-drinking (*n* = 5) participants who were purposively selected to represent a wide range of drinking levels, ages, and occupations were asked what they perceived to be the positive or negative consequences of various WHO-recommended strategies. A variety of answers were collected, for example, “it will be bad for business and the economy”, “it will negatively affect Hong Kong's lifestyle”, “it will improve image of Hong Kong as a healthy city”, “it will make Hong Kong oppressive”, “it will save money on law enforcement”. Formulated based on these findings and pilot survey results, the instrument asks whether the respondents agree (yes/no) that implementing the various strategies would: 1) reduce alcohol-related harms in Hong Kong, 2) hurt business and the economy, 3) negatively affect local lifestyles, 4) infringe on economic freedom (applicable to marketing restrictions only), or 5) other.

### Statistical Analysis

Principal component analysis (PCA) was conducted to identify the general pattern of the support for 17 items of alcohol policies recommended by the WHO, by linearly combining them into a smaller set of principal components (PCs) with minimum loss of information. The Kaiser-Meyer-Okin (KMO) measure was used to test the adequacy of sample size, which is indicated by a KMO value larger than 0.5. Bartlett's sphericity test was conducted to examine whether the correlation between variables is satisfactory for PCA. PCs with eigenvalues larger than one were retained for further analyses according to Kaiser's rule. Orthogonal varimax rotation was used to identify the group of policy items that are highly correlated with only one single PC. Given the sample size (*n* = 4,000), PC loadings of 0.3 or higher were considered significant for interpretative purposes (43). The analysis generated a score for each identified PC for each participant, and this score was used as the indicator of support for different alcohol policy categories. To test the robustness of PCA results, sensitivity analysis was conducted: 1) because the assumption of PCA that the total variance is equal to common variance may not be fully met, we conducted a maximum likelihood factor analysis as an alternative approach for data reduction; 2) simple unit weighting was used to generate factor scores, calculated by the sum of items that load on a factor, and this factor score was used as the alternative indicator of support for different alcohol policy categories in multivariable models; 3) Cronbach's alpha was calculated to test the coherency of items within each factor.

**Figure 1:**
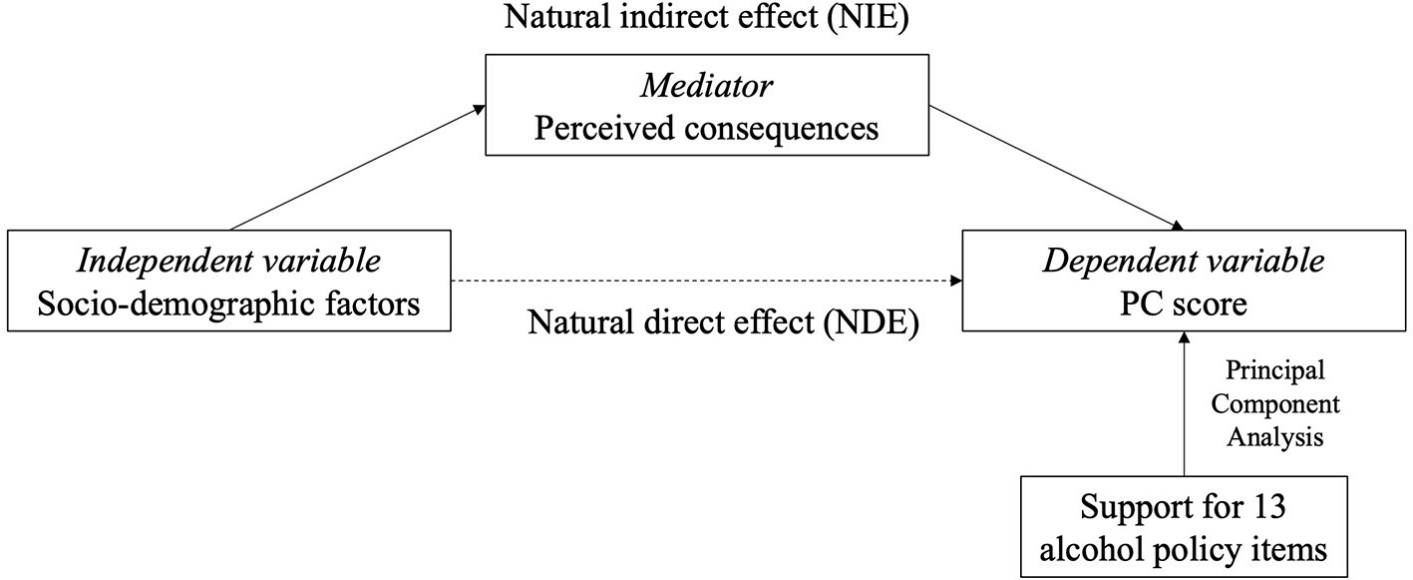
Conceptual model. Indirect relationship of socio-demographic factors with policy support (measured by PC scores) through mediators (perceived consequences). PC, principal component.

Univariable linear regression was conducted separately for each identified principal component to examine the association between demographics, attitudinal, and drinking-related factors and PC score. Variables that were significant at *p* <0.20 level were further included in two sets of stepwise multivariable linear regression models (44). The first model included only socio-demographic candidate variables; the second “all variables model” included attitudinal and drinking-related candidate variables, with significant demographic factors controlled for. Coefficients (β) were reported with 95% confidence intervals (CIs). Statistical significance was set at *p* < 0.05. Due to a high percentage of missing data for income (19.2% of the sample), an “unknown” indicator variable was included as one of the income categories in the multivariable analyses. Statistical tests indicated that respondents with missing data on income were more likely to be young, highly educated, and employed (*p* < 0.05). Causal mediation analysis (45) was used to test the mediation effects of perceived consequences in any significant associations between the socio-demographic variables with the policy endorsement measured by PC scores (Figure 1). The total effect of socio-demographic factors on policy endorsement was decomposed into natural direct effect and natural indirect effect (mediation) through perceived consequences. All statistical analyses were performed using Stata 14.0 (Stata Corp LP, College Station, TX, USA).

## Results

This study sampled 1,737 male and 2,263 female Hong Kong residents. The demographic characteristics of the study sample (Table 1) were comparable to the Hong Kong Census population (46) in gender, age, and area of residence. However, our sample had a higher proportion of university-educated adults and a lower proportion of high-income individuals compared to the general population. Our overall sample had a higher proportion of housewives, which is likely due to the extensive calling hours of the telephone survey. The proportions of ever drinker and past-year drinker were 57.5 and 44.3% among males and 36.7 and 28.7% among females. Of all respondents, 7.2% were past-month binge drinkers and 6.5% drank at least once a week.

### Acceptability of the WHO “Best Buys” and Other Recommendations

Among all the WHO “Best Buys”, the most publicly-supported strategies were introducing 5–10% beer/wine taxes (68.7%), restricting alcohol retail hours (51.9%), and limiting bar service hours (44.5%) (Table 2). Among other WHO-recommended strategies, there was very high public support for interventions such as youth-oriented education (96.1%), enforcement of random breath tests (92.4%), and age verification for alcohol purchases at stores (83.6%) and at bars/restaurants (81.4%). By contrast, the least popular measures were sponsorship bans on high-publicity events such as concerts and sporting events (19.5%) and bans on public drinking events (e.g., Hong Kong Wine Festival) (17.7%). Past-year abstainers are significantly more supportive of all strategies than past-year drinkers (*p* < 0.05).

### Socio-Demographic and Attitudinal Factors Associated With Strategy Endorsement

The result of Bartlett's sphericity test (*p* < 0.001) suggested that the correlation between various policy items was satisfactory, and a KMO value of 0.77 indicated an adequate study sample size for PCA. Following Kaiser's rule, six PCs were retained, which explained a total of 71.9% of the variance. All of the 17 policy items assessed had at least one rotated PC loading above 0.3 and therefore none were excluded from the model (43). The PC structure and rotated PC loadings are displayed in Supplementary Table 1. Of the 17 policy items, 13 were reduced into five components which were labeled as “Age Verification for Young Drinkers”, “Taxation and Pricing”, “Availability Restriction”, “Advertising Restriction”, and “Sponsorship and Events Restriction”. The remaining four policy items (enforcing random breath test, mandating health warning labels, promoting services for alcoholics, and promoting alcohol-related education), which were collapsed under the same “Other policies” component, did not appear to represent a conceptually coherent factor and none of these items are part of the WHO “Best Buys”. This component was therefore excluded from further analysis. Subsequently, we reran the PCA after excluding these four policy items and confirmed that the same five PCs above were identified (Bartlett's sphericity test *p* < 0.001; KMO = 0.76; 78.4% of variance explained) (Table 3). The determinants for each of these five PCs were examined using univariable and multivariable regression analyses where PC score was the dependent variable (Table 4).

**Table 3:** Principal component analysis of 13 policy items by five principal components (PCs)–rotated PC loadings.

**Alcohol policy item**	**Age verification for young drinkers**	**Taxation and pricing**	**Availability restriction**	**Advertisement restriction**	**Sponsorship and events restriction**
Requesting ID at stores	**0.706**	0.001	0.008	−0.002	−0.002
Requesting ID at bars and restaurants	**0.706**	0.001	−0.003	0.007	0.001
Implementing a moderate beer and wine tax	−0.001	**0.719**	−0.016	0.042	−0.185
Re-introducing a heavy 30% beer and wine tax	−0.054	**0.373**	0.094	0.153	0.060
Setting a minimum alcohol price	0.027	**0.581**	−0.017	−0.129	0.275
Convenience stores not being permitted to sell alcohol after a certain time	0.012	−0.005	**0.601**	0.003	−0.033
Introducing “Last Order” in bars	0.005	−0.012	**0.585**	0.031	−0.054
Limiting the number of alcohol serving establishments outside of the tourist areas	−0.014	−0.008	**0.535**	−0.054	0.101
Banning large alcohol advertisements on public billboards and public transport	0.001	0.009	−0.004	**0.592**	−0.010
Banning all alcohol advertising on TV, radio, and magazines	0.001	0.007	−0.002	**0.597**	−0.031
Greater social media regulation of alcohol advertisements	0.013	−0.042	−0.005	**0.497**	0.104
Banning alcohol event sponsorship	0.006	−0.054	−0.015	0.019	**0.661**
Restricting high publicity drinking events	−0.010	−0.013	0.006	0.003	**0.651**

*PC, principal component. Bold values indicate the policy items loaded on each principal component*.

**Table 4:** Socio-demographic, attitudinal, and drinking-related factors associated with endorsement of alcohol harms reduction strategies measured by PC scores.

	**Age verification for**		**Taxation and pricing**		**Availability restriction**		**Advertising restriction**		**Sponsorship and events**	
	**young drinkers**								**restriction**	
	**Model 1^**a**^**	**Model 2^**b**^**	**Model 1^**a**^**	**Model 2^**b**^**	**Model 1^**a**^**	**Model 2^**b**^**	**Model 1^**a**^**	**Model 2^**b**^**	**Model 1^**a**^**	**Model 2^**b**^**
***Socio-demographic factors***	***Coef. (95% CI)***	***Coef. (95% CI)***	***Coef. (95% CI)***	***Coef. (95% CI)***	***Coef. (95% CI)***	***Coef. (95% CI)***	***Coef. (95% CI)***	***Coef. (95% CI)***	***Coef. (95% CI)***	***Coef. (95% CI)***
Gender										
Male	Ref.	Ref.	Ref.	Ref.	Ref.	Ref.	Ref.	Ref.	Ref.	Ref.
Female	0.32 (0.24, 0.41)^†^	0.29 (0.21, 0.38)^†^	0.15 (0.07, 0.22)^†^	0.11 (0.04, 0.18)^§^	0.34 (0.25, 0.44)^†^	0.34 (0.25, 0.44)^†^	0.32 (0.22, 0.42)^†^	0.24 (0.14, 0.34)^†^	0.25 (0.17, 0.34)^†^	0.19 (0.11, 0.28)^†^
Age										
18–34	Ref.	Ref.	Ref.	Ref.	1.00	Ref.	Ref.	Ref.	Ref.	Ref.
35–54	0.11 (0.01, 0.22)*	0.05 (−0.05, 0.16)	0.14 (0.02, 0.36)*	0.09 (−0.01, 0.19)	0.39 (0.28, 0.51)^†^	0.25 (0.13, 0.37)^†^	−0.24 (−0.35, −0.13)^†^	−0.11 (−0.22, 0.01)	−0.06 (−0.17, 0.05)	−0.11 (−0.22, −0.004)*
55–74	0.40 (0.29, 0.51)^†^	0.30 (0.18, 0.41)^†^	0.28 (0.14, 0.42)^†^	0.24 (0.14, 0.35)^†^	0.63 (0.50, 0.76)^†^	0.45 (0.32, 0.58)^†^	−0.54 (−0.10, −0.07)*	−0.49 (−0.96, −0.03)*	0.27 (0.16, 0.39)^†^	0.21 (0.09, 0.33)^§^
Marital status										
Currently married	–	–	Ref.	–	–	–	–	–	–	–
Single, never married	–	–	−0.11 (−0.22, 0.00)*	–	–	–	–	–	–	–
Divorced/ separated/widowed	–	–	−0.27 (−0.61, 0.07)	–	–	–	–	–	–	–
Education										
Secondary or less	–	–	Ref.	Ref.	–		–	–	–	–
Upper secondary Non-degree	–	–	−0.21 (−0.35, −0.06)^§^	−0.19 (−0.34, −0.05)^§^	–		–	–	–	–
University or above	–	–	−0.12 (−0.21, −0.02)*	−0.09 (−0.18, −0.01)*	–		–	–	–	–
Employment										
Unemployed	–	–	Ref.	–	Ref.	Ref.	Ref.	Ref.	Ref.	Ref.
Employed	–	–	−0.13 (−0.22, −0.04)^§^	–	−0.42 (−0.53, −0.30)^†^	−0.29 (−0.40, −0.18)^†^	−0.35 (−0.46, −0.24)^†^	−0.27 (−0.38, −0.16)*	−0.33 (−0.43, −0.23)^†^	−0.26 (−0.36, −0.16)^†^
Monthly household income										
<25,000 HKD	–	–	Ref.	Ref.	Ref.	Ref.	Ref.	Ref.	Ref.	Ref.
25,000–49,999 HKD	–	–	0.03 (−0.07, 0.12)	0.03 (−0.06, 0.12)	−0.15 (−0.27, −0.03)*	−0.11 (−0.23, 0.01)	−0.17 (−0.30, −0.05)^§^	−0.13 (−0.25, −0.01)*	−0.22 (−0.33, −0.11)^†^	−0.20 (−0.31, −0.10)^†^
≥50,000 HKD	–	–	−0.27 (−0.40, −0.14)^†^	−0.22 (−0.35, −0.09)^§^	−0.07 (−0.23, 0.10)	−0.01 (−0.16, 0.16)	−0.25 (−0.42, −0.08)^§^	−0.10 (−0.27, 0.07)	0.15 (0.01, 0.30)*	0.14 (−0.01, 0.29)
Unknown	–	–	−0.14 (−0.25, −0.04)^§^	−0.16 (−0.26, −0.05)^§^	−0.01 (−0.25, 0.02)	−0.14 (−0.27, −0.01)*	−0.37 (−0.51, −0.23)^†^	−0.38 (−0.52, −0.24)^†^	−0.23 (−0.36, −0.11)^†^	−0.24 (−0.36, −0.12)^†^
***Attitudinal factors***										
Alcohol use is a public health issue		0.25 (0.16, 0.34)^†^		0.14 (0.07, 0.22)^†^		0.35 (0.25, 0.44)^†^		–		0.42 (0.33, 0.51)^†^
Habitual drinking is bad for health		0.43 (0.26, 0.59)^†^		0.53 (0.39, 0.67)^†^		0.34 (0.16, 0.51)^†^		0.42 (0.23, 0.61)^†^		0.17 (0.01, 0.33)*
Occasional drinking is good for health		0.17 (0.83, 0.26)^†^		–		−0.11 (−0.21, −0.01)*		−0.12 (−0.22, −0.01)*		–
Not knowing drink is bad for business		–		–		–		–		−0.10 (−0.19, −0.02)*
Drinking has noticeable social benefits		0.17 (0.06, 0.27)^§^		–		−0.18 (−0.29, −0.07)^§^		−0.47 (−0.58, −0.35)^†^		−0.18 (−0.29, −0.08)^§^
***Drinking-related factors***										
Past-year drinking										
Abstainer		Ref.		Ref.		Ref.		Ref.		Ref.
Non-binge drinker		0.01 (−0.1, 0.10)		−0.30 (−0.39, −0.22)^†^		−0.42 (−0.53, −0.31)^†^		−0.31 (−0.43, −0.19)^†^		−0.12 (−0.22, −0.02)*
Binge drinker		−0.58 (−0.75, −0.40)^†^		−0.60 (−0.74, −0.45)^†^		−0.86 (−1.05, −0.67)^†^		−0.49 (−0.69, −0.39)^†^		−0.22 (−0.40, −0.05)*

*p < 0.05.

*^§^p < 0.01.*

*^†^p < 0.001.*

*^a^Model 1 included socio-demographic factors.*

*^b^Model 2 (“All Variable Model”) further included attitudinal, drinking-related factors based on Model 1; AOR, adjusted odds ratio derived from stepwise multivariable logistic regression using univariable significant level p < 0.20 as candidate variable*.

Results of the socio-demographic regression model showed that female respondents and older adults were significantly more supportive of all policy categories than males and younger adults. Adults who were unemployed were significantly more supportive of all policy categories except age verification for young drinkers. Highly educated respondents were less supportive of taxation and pricing policies. High income was positively associated with endorsement of sponsorship restriction, but negatively associated with support for advertising restriction and taxation and pricing measures. Having dependent children did not show any significant association with support for the five policy categories.

The full regression model suggested that after adjusting for socio-demographic variables, those perceiving alcohol as a local public health issue were more likely to endorse all policy categories except advertising restriction. Concern for drinking's health harms was significantly associated with higher endorsement of all policy categories. On the other hand, those who believed in the health benefits of occasional drinking showed greater support for age verification but lower support for availability and advertising restrictions. Belief that drinking is advantageous in business undermined the support for sponsorship and event restrictions whereas perception of alcohol's social benefits predicted lower endorsement of availability and marketing restrictions. Drinkers, particularly binge drinkers, were significantly less supportive of most policy categories than past-year abstainers.

In sensitivity analysis, maximum likelihood factor analysis retained 10 policies items which loaded on four factors labeled “Age Verification for Young Drinkers”, “Availability Restriction”, “Advertisement Restriction”, and “Sponsorship and Events Restriction” (Supplementary Table 2). Although the taxation and pricing strategies were removed from the model, the four remaining factors were consistent with components from PCA. The Cronbach's alpha coefficient of four subscales were >0.80, suggesting satisfactory internal reliability of the identified factors. We tried conducting unit weighting for factor scores, and the significant socio-demographic, attitudinal, and drinking-related factors identified by multivariable models were not substantively different from the original findings for these four policy categories.

### Perceived Consequences of WHO-Recommended Strategies

Table 5 describes the possible positive and negative consequences of implementing the five major policy categories as perceived by the participants. These results and findings of acceptability level altogether demonstrated a general pattern that the perceived effectiveness in mitigating alcohol-related harms is counterbalanced by public concerns about lifestyle infringements and detriment to the local economy. The majority of respondents believed that age verification for young drinkers (82.4%), restriction of alcohol availability (68.9%), and alcohol price increases (57.9%) would be able to reduce alcohol-related problems in Hong Kong (Table 5). Restriction of alcohol availability was perceived to be the most likely to hurt local business and economy (48.5%), followed by sponsorship and event bans (46.2%). Almost half of the respondents agreed that bans of alcohol sponsorship (44.7%) and advertising (40.3%) would be an infringement on economic freedoms. Alcohol availability restrictions (40.9%) and tax and price increases (35.7%) were perceived to be the most likely to undermine local lifestyles. Past-year abstainers had higher expectancies of strategy effectiveness in reducing harms whereas drinkers expressed more concerns on negative consequences on the economy, lifestyle, and economic freedom.

**Table 5:** Perceived consequences of implementing alcohol harms reduction strategies among participants and by past-year drinking status (*n* = 4,000).

**Consequences of strategies**	**Endorsing respondents %**			
	**Past-year drinkers, *n* = 1,418**	**Past-year abstainers, *n* = 2,582**		**Overall, *n* = 4,000**
***Drinking Age Verification for Young Drinkers***	***% (95% CI)***	***% (95% CI)***	***P(** **χ^2^)***	***% (95% CI)***
Will reduce underage drinking	77.4 (75.1–79.5)	85.0 (83.6–86.4)	<0.001	82.4 (81.1–83.5)
Is bad for business and economy	28.7 (26.3–31.0)	28.4 (26.7–30.2)	0.99	28.5 (27.1–29.9)
Will negatively affect local lifestyle	26.9 (24.6–29.2)	26.8 (25.1–28.5)	0.82	27.0 (25.6–28.4)
***Tax and Price Increases***				
Will reduce alcohol–related harms in Hong Kong	51.5 (48.9–54.1)	61.0 (59.1–62.9)	<0.001	57.9 (56.3–59.4)
Is bad for business and economy	48.3 (45.7–50.9)	37.9 (36.0–39.8)	<0.001	41.7 (40.1–43.2)
Will negatively affect local lifestyle	40.3 (37.7–42.8)	33.2 (31.4–35.0)	<0.001	35.7 (34.3–37.2)
***Restriction of Availability***				
Will reduce alcohol–related harms in Hong Kong	61.5 (58.9–64.0)	72.6 (70.9–74.3)	<0.001	68.9 (67.4–70.3)
Is bad for business and economy	52.7 (49.8–55.7)	45.0 (44.1–47.0)	<0.001	48.5 (47.0–50.0)
Will negatively affect local lifestyle	48.8 (46.2–51.4)	36.2 (34.4–38.1)	<0.001	40.9 (39.4–42.4)
***Advertisement Bans***				
Will reduce alcohol-related harms in Hong Kong	31.5 (29.2–34.0)	35.5 (33.2–36.9)	0.01	34.1 (32.6–35.6)
Is bad for business and economy	39.2 (36.7–41.8)	31.0 (29.2–32.8)	<0.001	34.0 (32.5–35.5)
Will negatively affect local lifestyle	25.8 (23.6–28.1)	24.9 (23.2–26.6)	0.63	25.3 (24.0–26.7)
Will infringe economic freedom	47.0 (44.4–49.6)	37.6 (36.5–39.9)	<0.001	40.3 (38.8–41.8)
***Sponsorships and Events Bans***				
Will reduce alcohol-related harms in Hong Kong	30.5 (28.2–33.0)	38.0 (36.2–39.9)	<0.001	35.6 (34.1–37.1)
Is bad for business and economy	52.4 (49.8–55.0)	42.5 (40.6–44.4)	<0.001	46.2 (44.6–47.7)
Will negatively affect local lifestyle	30.5 (28.2–33.0)	29.0 (27.2–30.8)	0.39	29.7 (28.3–31.1)
Will infringe economic freedom	51.7 (49.1–54.3)	40.7 (38.8–42.6)	<0.001	(43.1–46.2)

### Mediation Effects of Consequence Perception on Strategy Endorsement

Based on the results from multivariable regression (Table 4), the perceived consequences were examined as potential mediators in causal mediation analysis (45). Partial mediating effects were found in the association between some significant socio-demographic factors and the endorsement of different policies (Table 6). The results showed that women, older people, and unemployed people were more supportive of various alcohol policies partially because of greater belief that these restrictions would reduce alcohol-related problems (mediation proportion range: 11.7–31.3%, *p* < 0.05). By contrast, those with high household income were less supportive partially due to worries for negative impacts on local business, lifestyles, and economic freedom (mediation proportion range: 7.2–28.3%, *p* < 0.05). The complete results of mediation analysis are presented in Supplementary Table 3.

**Table 6:** Mediational effects of perceived consequences in associations between socio-demographic factors and support for alcohol harms reduction strategies measured by PC scores, adjusted for other socio-demographic factors.

	**Age verification for**		**Taxation and pricing**		**Availability restriction**		**Advertising restriction**		**Sponsorship and events**	
	**young drinkers**								**restriction**	
**Mediational effects**	**NDE, NIE**	**%**	**NDE, NIE**	**%**	**NDE, NIE**	**%**	**NDE, NIE**	**%**	**NDE, NIE**	**%**
	**(coefficient)**	**mediated^**b**^**	**(coefficient)**	**mediated^**b**^**	**(coefficient)**	**mediated^**b**^**	**(coefficient)**	**mediate^**b**^**	**(coefficient)**	**mediated^**b**^**
***Mediators of female gender effect** ^***a***^*										
Reduces alcohol-related problems	0.21*, 0.08*	28.9%	0.11*, 0.02*	17.9%	0.33*, 0.11*	24.1%	0.23*, 0.10*	31.3%	0.18*, 0.07*	26.7%
Hurts local business and economy	NS	–	NS	–	0.43*, 0.01*	2.5%	NS	–	NS	–
Infringes on economic freedom							0.30*, 0.03*	8.7%	NS	–
***Mediators of older age effect** ^***a***^*										
Reduces alcohol-related problems	0.29*, 0.10*	24.7%	NS	–	0.50*, 0.07*	11.7%	0.27*, 0.07*	21.2%	0.37*, −0.02*	NA
Negatively affects lifestyles	NS	–	NS	–	NS	–	NS	–	0.37*, −0.02*	NA
***Mediators of employment effect** ^***a***^*										
Reduces alcohol-related problems	NC	–	NC	–	−0.35*, −0.05*	12.4%	NS	–	−0.37*, −0.06*	13.6%
Hurts local business and economy	NC	–	NC	–	NS	–	−0.36*, 0.03*	NA	NS	–
Negatively affects lifestyles	NC	–	NC	–	−0.43*, 0.01*	NA	−0.38*, 0.04*	NA	NS	–
***Mediators of high income effect** ^***a***^*										
Reduces alcohol-related problems	NC	–	NS	–	−0.35*, −0.05*	12.4%	−0.19*, −0.04*	17.0%	−0.08*, −0.04*	36.7%
Hurts local business and economy	NC	–	−0.11*, −0.01*	7.2%	NS	–	NS	–	−0.10*, −0.02*	13.8%
Negatively affects lifestyles	NC	–	−0.11*, −0.02*	12.9%	−0.43*, 0.01*	NA	−0.20*, −0.03*	13.4%	NS	–
Infringes on economic freedom							−0.16*, −0.06*	28.3%	−0.10*, −0.02*	16.1%

*p < 0.05.

*^a^Effect of independent variables: gender (male vs. female); age (18–34 years vs. 55–74 years); employment (unemployed vs. employed); income (<25,000 HKD vs. ≥50,000 HKD).*

*^b^% mediated=NIE/(NDE+NIE), NDE, natural direct effect. NIE, natural indirect effect; NA, not available; NS, the mediational effect is not significant; NC, causal medation analysis was not conducted due to Non-significant association between the socio-demographic factor and policy support*.

## Discussion

This population-based study was the first to examine the public acceptability of alcohol control policies and the associated factors in the China region. Our findings noted that among the WHO's “Best Buys” on alcohol taxation, availability, and advertising that are highly relevant to the current policy gap of Hong Kong, the most strongly supported were introduction of moderate alcohol taxes, restrictions on time of retail alcohol sales, and limiting bar service hours. Aside from the “Best Buys”, youth-oriented interventions such as age verification for young drinkers and education provisions are highly popular. This finding indicates, as in other countries, that interventions aimed at young people have much less public resistance (3). Reducing alcohol service hours and tax increases were previously found to be the least popular policies in England, (47) Canada, (26) Australia, (6) and Sweden (48) where varying levels of taxes and service hour restrictions already exist. The high levels of support for these measures in Hong Kong likely reflects the considerable lower prevalence of habitual drinking in Hong Kong (44.3% of males and 28.7% of women were past-year drinkers) as compared with most Western countries (1). The relatively high level of acceptance for a moderate 5–10% tax may also reflect the zero tax on beer and wine that is unique to Hong Kong. In our sample, however, past-year drinkers were consistently less likely to support stricter regulation. It is likely that drinkers, particularly heavy and frequent drinkers, perceive alcohol control measures as inconveniences and an infringement on their lifestyle. Compared with South Korea where alcohol policies are more lax than Western countries, Hong Kong residents were slightly more supportive for increasing taxes, restricting alcohol availability, and age verification for young drinkers, but are more resistance to marketing restriction (9). This may be related to the low prevalence of severe alcohol-related harms and pervasive laissez-faire economy among the population.

Our results indicate that the skepticism about policy effectiveness may be a major barrier to public support. In contrast to some Western countries where as much as half of the population supported various alcohol advertising restrictions (49–51), banning alcohol marketing, particularly sponsorships, was the least endorsed policy among Hong Kong residents. While age verification and restricting alcohol availability were seen as the most effective in alcohol harms reduction strategies, marketing restrictions were perceived as the least effective by the Hong Kong public. Our results are consistent with prior findings that the perceived effectiveness is strongly associated with public support for policy (7, 23). In general, the public acceptability in Hong Kong was greater for strategies that were perceived to be effective in mitigating alcohol-related problems while being unlikely to harm economic interests. Our results largely corroborate findings from previous international studies suggesting that females and older adults were consistently more supportive of stringent population-level alcohol policies (7, 9–11, 13–15). In our study, we noted that older people and women also had greater optimism about the policy's effectiveness in reducing alcohol-related harms which may also partially explain their greater endorsement of more stringent regulations. Other possible reasons for greater support among these population subgroups may be the higher likelihood of second-hand harms (harms due to others' drinking) among women (e.g., domestic violence, marital problems, caretaking of drinking family members) (3, 52, 53) and greater awareness of the health-related alcohol harms among older adults as compared to younger drinkers (3, 54). Our results showed that parents of minor were not more likely to support greater alcohol regulation. This finding may be explained by the comparatively low rate of binge drinking and severe alcohol-related harms (e.g., from traffic crashes and drinking events) among young people than in Western settings.

In addition to perceived effectiveness, perception of possible consequences of various strategies was associated with levels of public support. The public skepticism toward alcohol policies largely comes from concerns of possible negative consequences on local business and economy rather than infringements on individual civil liberties. Hong Kong has consistently ranked as the freest economy in the world (55) and the laissez-faire economic culture has long been supportive of international business and trade. The negative economic expectancies are more common among the younger generation, contributing to their unfavorable attitudes toward alcohol policies. In contrast to most developed Western countries, in Hong Kong, as in some other regions of Asia, the collective economic prosperity is often prioritized over public health interests when devising public policies, including alcohol policies (9, 56). Past research has suggested that East Asians tend to adopt a more collectivistic perspective than individualistic compared to norms in Western cultures (57–59). Hence, under such culture norm, the economic benefits of policies must be counterbalanced by pervasive social harms in order for a policy to be acceptable. Due to the low levels of automobile ownership and strictly restricted firearms, serious alcohol-related harms including interpersonal violence and traffic fatalities in Hong Kong are much more uncommon than in countries such as the US and UK (60–62). This could partially explain why there has not been strong advocacy for greater alcohol regulation. Although data on perceived consequences of implementing various alcohol policies are very scarce in East Asian region, it is likely that the acceptability of policy recommendations in other Asian cities rests in the perceived trade-off of benefits to the local economy vs. the pervasiveness of societal harms.

In addition to economic externalities, our findings suggest that beliefs of drinking's social benefits lowered support for greater regulation. Over the past two decades, the Hong Kong alcohol industry has strategically used mass media to shape the public perception that alcohol regulation would deprive ordinary drinkers of their enjoyment; health benefits of moderate drinking has also been strongly promoted (63). Until recently, there has been relatively less public health information on alcohol. Therefore, misconceptions about health effects of drinking are likely to be pervasive in the general public. In our sample, concerns about alcohol-related health harms generally predicted higher endorsement of WHO-recommended strategies, as noted elsewhere (23, 64). A media campaign that aims to promote public awareness of alcohol-related health and social harms and public health significance of effective countermeasures may be effective in shifting public opinion. It is critical to reinforce the belief on the effectiveness of strategies and dispel common misconceptions among the general public prior to the introduction of any new alcohol policy, particularly among men and young people.

There are limitations to this study. First, the study instrument was developed for assessment purposes and needs future validation. The findings are prone to information bias from self-reported data, but this is minimized by assuring anonymity in telephone interviews. Second, despite the high fixed landline penetration of Hong Kong (93%) (65), certain population subgroups such as individuals living in elderly homes, prisons, dorms, and vessels could not be reached by landlines. Although the response rate of our survey (62.5%) was typical as a telephone survey conducted in this region (37, 66), the over-representation of Non-working and higher educated population might reduce the generalisability of results. Nonetheless, a study from New Zealand concluded that public attitudes surveys may not be subject to strong Non-response biases (67). Third, only four attitudinal questions were included, and the mediating effects of perceived consequences have not been fully explored, which warrants further studies. Additionally, while we acknowledge that the assumption of PCA may not be met fully and the loading estimates may be biased upward compared to common factor analysis approaches, the findings are supported by consistent results from maximum likelihood factor analysis. PCA was chosen as the preferred method of data reduction because the identified components largely replicate the clustering of alcohol strategies that are put forth in alcohol action plans and the PCA did not eliminate key WHO “Best Buys” strategies. Lastly, the cross-sectional study design precludes any conclusions of causality between attitudes toward alcohol and policy endorsement. To better inform policy making, future research is needed to acquire more knowledge on the complex association between alcohol policy, perceived effectiveness, and policy acceptability, and to increase the understanding of how these attitudes are formed.

In conclusion, this study demonstrates that public acceptability of alcohol regulation is strongly influenced by the local social context, as well as the perceptions of consequences vs. benefits of the various policies (68). Specifically, the main barriers to higher public support for increased alcohol regulation in Hong Kong are skepticism of alcohol policy effectiveness and the negative expectancies on the local economy rather than infringements on personal civil liberties. Further, in-depth investigation of these factors would, therefore, be highly informative in understanding public opinion before introducing a policy. To reduce potential barriers to implementing alcohol control strategies in a low alcohol consumption region, it is thereby important to educate the general public of alcohol-related harms and to strengthen their belief in the effectiveness of drinking countermeasures.

## Data Availability Statement

Data is available upon reasonable request by the author.

## Ethics Statement

Ethical approval was obtained from Ethics Committee of The Chinese University of Hong Kong. Written informed consent for participation was not required for this study in accordance with the national legislation and the institutional requirements.

## Author Contributions

The initial concept for this paper was developed by JK. Statistical analysis was completed by JY, TS, WG, and QF. The manuscript was written by JY, TS, JK, and DD. The editing process included JY, WG, DD, JK, and QF, and other authors approved the final manuscript. The overall process was led by JK. All authors contributed to the article and approved the submitted version.

## Funding

This work was supported by the Hong Kong General Research Fund [grant number 14611417].

## Conflict of Interest

The authors declare that the research was conducted in the absence of any commercial or financial relationships that could be construed as a potential conflict of interest.

## Publisher's Note

All claims expressed in this article are solely those of the authors and do not necessarily represent those of their affiliated organizations, or those of the publisher, the editors and the reviewers. Any product that may be evaluated in this article, or claim that may be made by its manufacturer, is not guaranteed or endorsed by the publisher.

## References

[1] World Health Organization. Global Status Report On Alcohol And Health 2018. Geneva (2018).

[2] World Health Organization. Global Status Report On Noncommunicable Diseases 2010. Geneva (2011).

[3] Diepeveen S Ling T Suhrcke M Roland M Marteau TM. Public acceptability of government intervention to change health-related behaviours: a systematic review and narrative synthesis. BMC Public Health. (2013) 13:756. 10.1186/1471-2458-13-756 23947336 PMC3765153

[4] Greenfield TK Ye Y Giesbrecht N. Alcohol policy opinions in the United States over a 15-year period of dynamic per capita consumption changes: implications for today's public health practice. Contemp Drug Probl. (2007) 34:649–80. 10.1177/009145090703400408

[5] Babor TF Caetano R Casswell S Edwards G Giesbrecht N Graham K. Alcohol: No Ordinary Commodity: Research and Public Policy Oxford: Oxford University Press (2010).

[6] Tobin C Moodie AR Livingstone C. A review of public opinion towards alcohol controls in Australia. BMC Public Health. (2011) 11:58. 10.1186/1471-2458-11-58 21272368 PMC3048532

[7] de Visser RO Hart A Abraham C Memon A Graber R Scanlon T. Which alcohol control strategies do young people think are effective? Drug Alcohol Rev. (2014) 33:144–51. 10.1111/dar.12109 24428843

[8] Parry CDH Trangenstein P Lombard C Jernigan DH Morojele NK. Support for alcohol policies from drinkers in the City of Tshwane, South Africa: data from the international alcohol control study. Drug Alcohol Rev. (2018) 37:S210–7. 10.1111/dar.12554 28493419 PMC5969057

[9] Seo S Chun S Newell M Yun M. Korean public opinion on alcohol control policy: A cross-sectional International Alcohol Control study. Heal Policy. (2015) 119:33–43. 10.1016/j.healthpol.2014.10.016 25442376

[10] Cook M Livingston M Vally H Callinan S. Australians' support for alcohol price-based policies. Int J Drug Policy. (2020) 85:102924. 10.1016/j.drugpo.2020.102924 32911321

[11] van der Sar R Brouwers EPM van de Goor IAM Garretsen HFL. The opinion of adolescents and adults on Dutch restrictive and educational alcohol policy measures. Health Policy. (2011) 99:10–6. 10.1016/j.healthpol.2010.06.025 20674060

[12] Dekker MR Jones A Maulik PK Pettigrew S. Public support for alcohol control initiatives across seven countries. Int J Drug Policy. (2020) 82:102807. 10.1016/j.drugpo.2020.102807 32526605

[13] Greenfield TK Karriker-Jaffe KJ Giesbrecht N Kerr WC Ye Y Bond J. Second-hand drinking may increase support for alcohol policies: new results from the 2010 National Alcohol Survey. Drug Alcohol Rev. (2014) 33:259–67. 10.1111/dar.12131 24761758 PMC4024451

[14] Karlsson D Holmberg S Weibull L. Solidarity or self-interest? Public opinion in relation to alcohol policies in Sweden. Nord Stud Alcohol Drugs. (2020) 37:105–21. 10.1177/1455072520904644 32934597 PMC7434170

[15] Sharp CA Bellis MA Hughes K Ford K di Lemma LCG. Public acceptability of public health policy to improve population health: a population-based survey. Heal Expect. (2020) 23:802–12. 10.1111/hex.13041 32329938 PMC7495082

[16] van Hoof JJ Gosselt JF de Jong MDT. Determinants of parental support for governmental alcohol control policies. Health Policy. (2010) 97:195–201. 10.1016/j.healthpol.2010.05.007 20627439

[17] Ialomiteanu AR Giesbrecht N Adlaf EM Irving H Paglia-Boak A Rehm J. An exploratory approach to analyzing alcohol control policy opinions held by Ontario adults. Int J Environ Res Public Heal. (2010) 7:7. 10.3390/ijerph7030827 20617006 PMC2872315

[18] Storvoll EE Moan IS Rise J. Predicting attitudes toward a restrictive alcohol policy: using a model of distal and proximal predictors. Psychol Addict Behav. (2015) 29:492–9. 10.1037/adb0000036 25347014

[19] van der Sar R Storvoll EE Brouwers EPM van de Goor LAM Rise J Garretsen HFL. Dutch and Norwegian support of alcohol policy measures to prevent young people from problematic drinking: a cross-national comparison. Alcohol Alcohol. (2012) 47:479–85. 10.1093/alcalc/ags032 22459020

[20] Holmila M Mustonen H Sterberg E Raitasalo K. Public opinion and community-based prevention of alcohol-related harms. Addict Res Theory. (2009) 17:360–71. 10.1080/16066350902770425

[21] Buykx P Gilligan C Ward B Kippen R Chapman K. Public support for alcohol policies associated with knowledge of cancer risk. Int J Drug Policy. (2015) 26:371–9. 10.1016/j.drugpo.2014.08.006 25217801

[22] Paglia A Room R. Expectancies about the effects of alcohol on the self and on others as determinants of alcohol policy attitudes. J Appl Soc Psychol. (1999) 29:2632–51. 10.1111/j.1559-1816.1999.tb00129.x

[23] Storvoll EE Rossow I Rise J. Changes in attitudes towards restrictive alcohol policy measures: The mediating role of changes in beliefs. J Subst Use. (2014) 19:38–43. 10.3109/14659891.2012.728671 24719564 PMC3971770

[24] Callinan S Room R Livingston M. Changes in Australian attitudes to alcohol policy: 1995–2010. Drug Alcohol Rev. (2014) 33:227–34. 10.1111/dar.12106 24372933

[25] Lonsdale AJ Hardcastle SJ Hagger MS. A minimum price per unit of alcohol: a focus group study to investigate public opinion concerning UK government proposals to introduce new price controls to curb alcohol consumption. BMC Public Health. (2012) 12:1023. 10.1186/1471-2458-12-1023 23174016 PMC3740777

[26] Giesbrecht N Ialomiteanu A Anglin L Adlaf E. Alcohol marketing and retailing: public opinion and recent policy developments in Canada. J Subst Use. (2007) 12:389–404. 10.1080/14659890701262189

[27] Nicholls JQ. Liberties and licences: alcohol in liberal thought. Int J Cult Stud. (2006) 9:131–151. 10.1177/1367877906064027

[28] Prichard J Matthews A Bruno R Rayment K James H. Detouring civil liberties? drug-driving laws in Australia. Griffith Law Rev. (2010) 19:330–49. 10.1080/10383441.2010.10854679

[29] Freedom House,. Countries Territories. (2021). Available online at: https://freedomhouse.org/countries/freedom-world/scores (accessed Jun 20, 2021).

[30] Department of Health - The Government of Hong Kong SAR. Alcohol Consumption Per Capita in Hong Kong. (2020). Available online at: https://www.change4health.gov.hk/en/alcohol_aware/figures/alcohol_consumption/index.html (accessed Apr 23, 2020).

[31] Higuchi S Matsushita S Maesato H Osaki Y. Japan: alcohol today. Addiction. (2007) 102:1849–62. 10.1111/j.1360-0443.2007.01902.x 17680852

[32] Kim MK Ko MJ Han JT. Alcohol consumption and mortality from all-cause and cancers among 1.34 million Koreans: the results from the Korea national health insurance corporation's health examinee cohort in 2000. Cancer Causes Control. (2010) 21:2295–302. 10.1007/s10552-010-9656-9 20941640

[33] Cochrane J Chen H Conigrave KM Hao W. Alcohol use in China. Alcohol Alcohol. (2003) 38:537–42. 10.1093/alcalc/agg111 14633640

[34] Yu J Sumerlin TS Goggins WB Dong D Chung RY Kim JH. First- and second-hand alcohol-related harms among urban Chinese: a population-based study from Hong Kong. Drug Alcohol Rev. (2022) 41:208–20. 10.1111/dar.13339 34184790

[35] Legislative Council - The Government of Hong Kong SAR. Dutiable Commodities (Amendment) Bill 2008. Legislative Council - The Government of Hong Kong SAR (2008).

[36] Hong Kong Trade Development Council. Wine Industry in Hong Kong. Hong Kong Trade Development Council (2017).

[37] Chung VCH Yip BHK Griffiths SM Yu ELM Kim JH Tam WWS. The impact of cutting alcohol duties on drinking patterns in Hong Kong. Alcohol Alcohol. (2013) 48:720–8. 10.1093/alcalc/agt065 23825091

[38] Kim JH Chan KWC Chow JKW Fung KP Fong BYF Cheuk KK. University binge drinking patterns and changes in patterns of alcohol consumption among Chinese undergraduates in a Hong Kong university. J Am Coll Heal. (2009) 58:255–65. 10.1080/07448480903295318 19959440

[39] Liquor Licensing Board,. List of Licensed Liquor Premises. (2020). Available online at: https://www.fehd.gov.hk/english/LLB_web/premis.html (accessed May 8, 2020).

[40] Legislative Council Hong Kong Government. Dutiable Commodities (Amendment) Ordinance 2018. Legislative Council Hong Kong Government (2018).

[41] Kim JH Lee S Chan KWC Lau J Tsang A Griffiths SM. A population-based study on the prevalence and correlates of drinking and driving in Hong Kong. Accid Anal Prev. (2010) 42:994–1002. 10.1016/j.aap.2009.12.001 20441805

[42] Department of Health - The Government of Hong Kong. Alcohol Screening and Brief Intervention - A Guide for Use in Primary Care. Hong Kong: Department of Health - The Government of Hong Kong (2017).

[43] Hair J Black W Babin B Anderson R. Multivariate Data Analysis. 6th ed. Harlow: Pearson (2010).

[44] Hosmer DW Lemeshow S Sturdivant RX. Applied Logistic Regression. 3rd ed. Hoboken, NJ: John Wileys & Sons, Inc. (2013). p. 91.

[45] Vanderweele TJ Vansteelandt S. Odds ratios for mediation analysis for a dichotomous outcome. Am J Epidemiol. (2010) 172:1339–48. 10.1093/aje/kwq332 21036955 PMC2998205

[46] Census Statistics Department - The Government of Hong Kong SAR. 2016 Population By-Census. (2018). Available online at: https://www.censtatd.gov.hk/hkstat/sub/sp459.jsp (accessed Sep 13, 2019).

[47] Bates S Holmes J Gavens L de Matos EG Li J Ward B. Awareness of alcohol as a risk factor for cancer is associated with public support for alcohol policies. BMC Public Health. (2018) 18:1–11. 10.1186/s12889-018-5581-8 29866082 PMC5987582

[48] Wallin E Andréasson S. Public opinion on alcohol service at licensed premises: a population survey in Stockholm, Sweden 1999–2000. Heal Policy. (2005) 72:265–78. 10.1016/j.healthpol.2004.09.001 15862635

[49] Moskalewicz J Wieczorek Ł Karlsson T Österberg E. Social support for alcohol policy: literature review. Drugs Educ Prev Policy. (2013) 20:361–74. 10.3109/09687637.2012.687794

[50] Greenfield TK Ye Y Giesbrecht NA. Views of alcohol control policies in the 2000 national alcohol survey: what news for alcohol policy development in the US and its States? J Subst Use. (2007) 12:429–45. 10.1080/14659890701262262

[51] Giesbrecht N Ialomiteanu A Anglin L. Drinking patterns and perspectives on alcohol policy: results from two Ontario surveys. Alcohol Alcohol. (2005) 40:132–9. 10.1093/alcalc/agh120 15582986

[52] Bellis MA Quigg Z Hughes K Ashton K Ferris J Winstock A. Harms from other people's drinking: an international survey of their occurrence, impacts on feeling safe and legislation relating to their control. BMJ Open. (2015) 5:e010112. 10.1136/bmjopen-2015-010112 26700293 PMC4691765

[53] Greenfield TK Karriker-Jaffe KJ Kaplan LM Kerr WC Wilsnack SC. Trends in alcohol's harms to others (AHTO) and co-occurrence of family-related AHTO: the four US national alcohol surveys, 2000–2015. Subst Abus Res Treat. (2015) 9:23–31. 10.4137/SART.S23505 26549971 PMC4624092

[54] Latimer WW Harwood EM Newcomb MD Wagenaar AC. Sociodemographic and individual predictors of alcohol policy attitudes: results from a US probability sample. Alcohol Clin Exp Res. (2001) 25:549–56. 10.1111/j.1530-0277.2001.tb02249.x 11329495

[55] Gwartney J Lawson R Hall J Murphy R Berggren N Mcmahon F. Economic Freedom of the World: 2020 Annual Report, Fraser Institute (2020).

[56] Guo X Huang Y. The development of alcohol policy in contemporary China. J Food Drug Anal. (2015) 23:19–29. 10.1016/j.jfda.2014.05.002 28911442 PMC9351742

[57] Kitayama S Park H Sevincer AT Karasawa M Uskul AK. A cultural task analysis of implicit independence: comparing North America, Western Europe, and East Asia. J Pers Soc Psychol. (2009) 97:236–55. 10.1037/a0015999 19634973

[58] Zou X Tam KP Morris MW Lee S lai Lau IYM Chiu C yue. Culture as common sense: perceived consensus vs. personal beliefs as mechanisms of cultural influence. J Pers Soc Psychol. (2009) 97:579–97. 10.1037/a0016399 19785480

[59] Yamagishi T Hashimoto H Schug J. Preferences vs. strategies as explanations for culture-specific behavior. Psychol Sci. (2008) 19:579–84. 10.1111/j.1467-9280.2008.02126.x 18578848

[60] Branas CC Han S Wiebe DJ. Alcohol use and firearm violence. Epidemiol Rev. (2016) 38:32–45. 10.1093/epirev/mxv010 26811427 PMC4762248

[61] Hong Kong Police Force Traffic Branch Headquarters. Traffic report - 2018. Hong Kong Police Force Traffic Branch Headquarters (2018).

[62] Department for Transport. Reported Road Casualties In Great Britain: 2019 Annual Report. Department for Transport (2020).

[63] Yoon S Lam T-H. The alcohol industry lobby and Hong Kong's zero wine and beer tax policy. BMC Public Health. (2012) 12:717. 10.1186/1471-2458-12-717 22935365 PMC3490743

[64] Slater MD Lawrence F Comello MLG. Media influence on alcohol-control policy support in the U.S. adult population: the intervening role of issue concern and risk judgments. J Health Commun. (2009) 14:262–75. 10.1080/10810730902805838 19440909 PMC2856665

[65] Office of the Communications Authority. Key Communications Statistics. (2018). Available online at: https://www.ofca.gov.hk/en/media_focus/data_statistics/key_stat/ (accessed May 28, 2018).

[66] Kim JH Lee S Chow J Lau J Tsang A Choi J. Prevalence and the factors associated with binge drinking, alcohol abuse, and alcohol dependence: a population-based study of Chinese adults in Hong Kong. Alcohol Alcohol. (2008) 43:360–70. 10.1093/alcalc/agm181 18230698

[67] Maclennan B Kypri K Langley J Room R. Non-response bias in a community survey of drinking, alcohol-related experiences and public opinion on alcohol policy. Drug Alcohol Depend. (2012) 126:189–94. 10.1016/j.drugalcdep.2012.05.014 22677457

[68] Hansis R. Social acceptability in anthropology and geography. In: Kelso W, Mark W. Brunson, Linda E. Kruger, Catherine B. Tyler, Susan A. SChroeder, editors. Defining Social Acceptability in Ecosystem Management: A Workshop Proceedings. Portland, Oregon: United States Department of Agriculture (1996). p. 37–47.

