# Supplementary material for: Selling World Health Organization's Alcohol “Best Buys” and Other Recommended Interventions in an Urban Chinese Population: Public Acceptability of Alcohol Harms Reduction Strategies in Hong Kong
Source: Front Public Health. 2022 Apr 21;10:855416. doi: 10.3389/fpubh.2022.855416 (PMC9068987; doi:10.3389/fpubh.2022.855416)
Supplement: Supplementary file 1 [file Table_1.DOCX]

**Supplementary File**

**Supplementary Table 1.** Principal component analysis of 17 policy items by six principal components (PCs) – rotated PC loadings.

| *Alcohol policy item* | *Age Verification for Young Drinkers* | *Taxation and Pricing* | *Availability Restriction* | *Advertisement Restriction* | *Sponsorship and Events Restriction* | *Others* |
| --- | --- | --- | --- | --- | --- | --- |
| Requesting ID at stores | **0.698** | 0.005 | 0.002 | -0.003 | -0.009 | -0.001 |
| Requesting ID at bars and restaurants | **0.701** | 0.008 | -0.006 | 0.008 | 0.005 | -0.011 |
| Implementing a moderate beer and wine  tax | -0.008 | **0.735** | -0.018 | 0.039 | -0.164 | 0.032 |
| Re-introducing a heavy 30% beer and  wine tax | -0.056 | **0.345** | 0.099 | 0.149 | 0.076 | 0.028 |
| Setting a minimum alcohol price | 0.056 | **0.563** | 0.003 | -0.122 | 0.283 | -0.048 |
| Convenience stores not being permitted  to sell alcohol after a certain time | 0.012 | 0.005 | **0.593** | 0.001 | 0.101 | 0.002 |
| Introducing “last order” in bars | 0.003 | -0.015 | **0.583** | 0.032 | -0.060 | -0.010 |
| Limiting the number of alcohol serving  establishments outside of the tourist  areas | -0.016 | -0.006 | **0.532** | -0.055 | 0.095 | -0.016 |
| Banning large alcohol advertisements on  public billboards and public transport | -0.0004 | 0.010 | -0.005 | **0.591** | -0.012 | 0.0002 |
| Banning all alcohol advertising on TV,  radio, and magazines | -0.001 | 0.009 | -0.002 | **0.597** | -0.032 | -0.001 |
| Greater social media regulation of  alcohol advertisements | 0.012 | -0.037 | -0.003 | **0.498** | 0.099 | -0.023 |
| Banning alcohol event sponsorship | 0.002 | -0.062 | -0.020 | 0.017 | **0.658** | 0.004 |
| Restricting high publicity drinking  events | -0.020 | -0.013 | -0.013 | 0.001 | **0.648** | -0.001 |
| Enforcement of current random breath  testing of drivers | -0.019 | 0.064 | -0.044 | 0.009 | -0.040 | **0.531** |
| More alcohol-related education especially  for young people | -0.063 | 0.039 | -0.044 | -0.024 | -0.021 | **0.579** |
| Mandatory health warning labels on  alcoholic beverages and advertisements | 0.083 | 0.089 | 0.077 | 0.041 | 0.062 | **0.376** |
| Increasing awareness of programmes like  AA for problem drinkers | 0.058 | -0.062 | 0.063 | -0.014 | 0.057 | **0.486** |

**Supplementary Table 2.** Factor analysis of 10 policy items by four factors – rotated factor loadings.

| *Alcohol policy item* | *Age Verification for Young Drinkers* | *Availability Restriction* | *Advertisement Restriction* | *Sponsorship and Events Restriction* |
| --- | --- | --- | --- | --- |
| Requesting ID at stores | **0.989** | 0.125 | 0.056 | 0.053 |
| Requesting ID at bars and restaurants | **0.879** | 0.122 | 0.077 | 0.051 |
| Convenience stores not being permitted  to sell alcohol after a certain time | 0.163 | **0.884** | 0.152 | 0.137 |
| Introducing “last order” in bars | 0.128 | **0.797** | 0.204 | 0.008 |
| Limiting the number of alcohol serving  establishments outside of the tourist  areas | 0.130 | **0.677** | 0.138 | 0.225 |
| Banning large alcohol advertisements on  public billboards and public transport | 0.063 | 0.144 | **0.938** | 0.166 |
| Banning all alcohol advertising on TV,  radio, and magazines | 0.064 | 0.144 | **0.901** | 0.168 |
| Greater social media regulation of  alcohol advertisements | 0.076 | 0.155 | **0.713** | 0.283 |
| Banning alcohol event sponsorship | 0.064 | 0.148 | 0.228 | **0.929** |
| Restricting high publicity drinking  events | 0.073 | 0.152 | 0.295 | **0.686** |

**Supplementary Table 3.** Mediational effects of perceived consequences in associations between socio-demographic factors and support for policy measured by PC scores, adjusted for other socio-demographic factors.

|  | *Age verification for*  *young drinkers* | | *Taxation and Pricing* | | *Availability Restriction* | | *Advertising Restriction* | | *Sponsorship and Events Restriction* | |
| --- | --- | --- | --- | --- | --- | --- | --- | --- | --- | --- |
|  | *NDE, NIE (coefficient, 95% CI)* | *% mediated ^b^* | *NDE, NIE (coefficient)* | *% mediated* | *NDE, NIE (coefficient)* | *% mediated* | *NDE, NIE (coefficient)* | *% mediated* | *NDE, NIE (coefficient)* | *% mediated* |
| *Mediators for the effect of female gender ^a^* | |  |  |  |  |  |  |  |  |  |
| Reduce alcohol-related problems | 0.21 (0.13, 0.29)*, 0.08 (0.05, 0.12)* | 28.9% | 0.11 (0.04, 0.19)*, 0.02 (0.01, 0.04)* | 17.9% | 0.33 (0.24, 0.42)*, 0.11 (0.07, 0.15)* | 24.1% | 0.23 (0.13, 0.32)*, 0.10 (0.07, 0.14)* | 31.3% | 0.18 (0.10, 0.26)*,  0.07 (0.03, 0.10)* | 26.7% |
| Hurt local business and economy | 0.30 (0.21, 0.39)*, 0.004 (-0.003, 0.004) | —— | 0.13 (0.06, 0.21)*, 0.01 (-0.002, 0.02) | —— | 0.43 (0.33, 0.53)*, 0.01 (0.001, 0.02)* | 2.5% | 0.32 (0.22, 0.42)*, 0.01 (-0.01, 0.03) | —— | 0.24 (0.15, 0.33)*,  0.01 (-0.001, 0.02) | —— |
| Negatively affect lifestyles | 0.30 (0.21, 0.39)*,  -0.003 (-0.01, 0.001) | —— | 0.14 (0.63, 0.21)*, 0.002 (-0.002, 0.006) | —— | 0.43 (0.33, 0.53)*, 0.01 (-0.01, 0.01) | —— | 0.31 (0.21, 0.41)*, 0.01 (-0.01, 0.03) | —— | 0.24 (0.16, 0.33)*,  0.01 (-0.003, 0.01) | —— |
| Infringe on economic freedom |  |  |  |  |  |  | 0.30 (0.20, 0.40)*, 0.03 (0.002, 0.05)* | 8.7% | 0.24 (0.15, 0.33)*,  0.01 (-0.003, 0.02) | —— |
| *Mediators for the effect of older age ^a^* | |  |  |  |  |  |  |  |  |  |
| Reduce alcohol-related problems | 0.29 (0.16, 0.43)*, 0.10 (0.04, 0.15)* | 24.7% | 0.26 (0.13, 0.38)*, 0.003 (-0.02, 0.03) | —— | 0.50 (0.34, 0.65)*, 0.07 (0.002, 0.13)* | 11.7% | 0.27 (0.13, 0.42)*, 0.07 (0.02, 0.13)* | 21.2% | 0.37 (0.22, 0.52)*,  -0.02 (-0.03, 0.001)* | NA |
| Hurt local business and economy | 0.40 (0.25, 0.55)*,  -0.01 (-0.02, 0.05) | —— | 0.26 (0.13, 0.39)*,  -0.00 (-0.003, 0.003) | —— | 0.57 (0.04, 0.74)*, -0.01 (-0.03, 0.02) | —— | 0.14 (-0.03, 0.32),  -0.02 (-0.07, 0.03) | —— | 0.37 (0.22, 0.52)*,  -0.02 (-0.05, 0.01) | —— |
| Negatively affect lifestyles | 0.40 (0.25, 0.55)*,  -0.003 (-0.02, 0.01) | —— | 0.26 (0.14, 0.39)*,  -0.003 (-0.01, 0.004) | —— | 0.56 (0.40, 0.73)*, 0.01 (-0.01, 0.03) | —— | 0.16 (-0.01, 0.33),  -0.04 (-0.08, -0.01) | —— | 0.37 (0.22, 0.52)*,  -0.02 (-0.03, 0.001)* | NA |
| Infringe on economic freedom |  |  |  |  |  |  | 0.11 (-0.06, 0.29), 0.001 (-0.06, 0.06) | —— | 0.35 (0.20, 0.50),  0.004 (-0.02, 0.03) | —— |
| *Mediators for the effect of being unmarried ^a^* | |  |  |  |  |  |  |  |  |  |
| Reduce alcohol-related problems | NC | —— | -0.10 (-0.19, -0.02)*, -0.02 (-0.04, 0.003) | —— | NC | —— | NC | —— | NC | —— |
| Hurt local business and economy | NC | —— | -0.12 (-0.21, -0.03)*, -0.006 (-0.01, 0.001) | —— | NC | —— | NC | —— | NC | —— |
| Negatively affect lifestyles | NC | —— | -0.11 (-0.20, -0.02)*, -0.01 (-0.02, 0.001) | —— | NC | —— | NC | —— | NC | —— |
| Infringe on economic freedom |  |  |  |  |  |  | NC | —— | NC | —— |
| *Mediators for the effect of high education ^a^* | |  |  |  |  |  |  |  |  |  |
| Reduce alcohol-related problems | NC | —— | -0.08 (-0.12, -0.03)*,  0.00 (-0.01, 0.01) | —— | NC | —— | NC | —— | NC | —— |
| Hurt local business and economy | NC | —— | -0.15 (-0.24, -0.06)*, -0.002 (-0.01, 0.003) | —— | NC | —— | NC | —— | NC | —— |
| Negatively affect lifestyles | NC | —— | -0.15 (-0.24, -0.06)*, -0.001 (-0.01, 0.01) | —— | NC | —— | NC | —— | NC | —— |
| Infringe on economic freedom |  |  |  |  |  |  | —— | —— | —— | —— |
| *Mediators for the effect of being employed ^a^* | |  |  |  |  |  |  |  |  |  |
| Reduce alcohol-related problems | NC | —— | NC | —— | -0.35 (-0.46, -0.27)*, -0.05 (-0.09, -0.01)* | 12.4% | -0.29 (-0.39, -0.18)*, -0.04 (-0.10, 0.07) | —— | -0.37 (-0.46, -0.28)*,  -0.06 (-0.09, -0.03)* | 13.6% |
| Hurt local business and economy | NC | —— | NC | —— | -0.42 (-0.53, -0.32)*, 0.01 (-0.001, 0.01) | —— | -0.36 (-0.47, -0.25)*, 0.03 (0.01, 0.05)* | NA | -0.43 (-0.53, -0.33)*, 0.001 (-0.002, 0.003) | —— |
| Negatively affect lifestyles | NC | —— | NC | —— | -0.43 (-0.53, -0.32)*, 0.01 (0.001, 0.02)* | NA | -0.38 (-0.49, 0.26)*, 0.04 (0.02, 0.07)* | NA | -0.43 (-0.53, -0.33)*,  -0.001 (-0.001, 0.006) | —— |
| Infringe on economic freedom |  |  |  |  |  |  | -0.34 (-0.45, -0.23)*, 0.01 (-0.02, 0.04) | —— | -0.43 (-0.53, -0.33)*,  -0.001 (-0.002, 0.003) | —— |
| *Mediators for the effect of high income ^a^* | |  |  |  |  |  |  |  |  |  |
| Reduce alcohol-related problems | NC | —— | -0.13 (-0.20, -0.06)*,  -0.01 (-0.01, 0.02) | —— | -0.35 (-0.46, -0.27)*, -0.05 (-0.09, -0.01)* | 12.4% | -0.19 (-0.28, -0.10)*, -0.04 (-0.08, -0.02)* | 17.0% | -0.08 (-0.15, 0.002)*,  -0.04 (-0.07, -0.02)* | 36.7% |
| Hurt local business and economy | NC | —— | -0.11 (-0.18, -0.04)*, -0.01 (-0.02, -0.002)* | 7.2% | -0.42 (-0.53, -0.32)*, 0.01 (-0.001, 0.01) | —— | -0.21 (-0.31, -0.12)*, -0.01 (-0.03, 0.01) | —— | -0.10 (-0.19, -0.02)*,  -0.02 (-0.03, -0.006)* | 13.8% |
| Negatively affect lifestyles | NC | —— | -0.11 (-0.18, -0.04)*, -0.02 (-0.03, -0.01)* | 12.9% | -0.43 (-0.53, -0.32)*, 0.01 (0.001, 0.02)* | NA | -0.20 (-0.29, -0.10)*, -0.03 (-0.05, -0.01)* | 13.4% | -0.11 (-0.19, -0.03)*,  -0.01 (-0.02, 0.001) | —— |
| Infringe on economic freedom |  |  |  |  |  |  | -0.16 (-0.26, -0.07)*, -0.06 (-0.09, -0.04)* | 28.3% | -0.10 (-0.18, -0.02)*,  -0.02 (-0.03, -0.01)* | 16.1% |

*p<0.05; NDE: natural direct effect; NIE: natural indirect effect; NA: not available; NC: the mediational analysis was not conducted due to non-significant association between the socio-demographic factor and policy support; PC: Principal Component; Effect of independent variables: gender (male vs. female); age (18-34 years vs. 55-74 years); marital status (married vs. single); education (secondary or less vs. university or above); employment (unemployed vs. employed); income (<25,000 HKD vs. ≥50,000 HKD); binge drinking (binge drinker vs. past-year abstainer). ^b^ calculated by NIE/(NDE+NIE).
